# Finite Element Simulation Model of Metallic Thermal Conductivity Detectors for Compact Air Pollution Monitoring Devices

**DOI:** 10.3390/s24144683

**Published:** 2024-07-19

**Authors:** Josée Mallah, Luigi G. Occhipinti

**Affiliations:** Electrical Engineering Division, Department of Engineering, University of Cambridge, Cambridge CB3 0FA, UK; jm2508@cam.ac.uk

**Keywords:** finite element modelling, gas sensors, simulation, thermal conductivity

## Abstract

Air pollution has been associated with several health problems. Detecting and measuring the concentration of harmful pollutants present in complex air mixtures has been a long-standing challenge, due to the intrinsic difficulty of distinguishing among these substances from interferent species and environmental conditions, both indoor and outdoor. Despite all efforts devoted by the scientific and industrial communities to tackling this challenge, the availability of suitable device technologies able to selectively discriminate these pollutants present in the air at minute, yet dangerous, concentrations and provide a quantitative measure of their concentrations is still an unmet need. Thermal conductivity detectors (TCDs) show promising characteristics that make them ideal gas sensing tools capable of recognising different gas analytes based on their physical fingerprint characteristics at the molecular level, such as their density, thermal conductivity, dynamic viscosity, and others. In this paper, the operation of TCD gas sensors is presented and explored using a finite element simulation of Joule heating in a sensing electrode placed in a gas volume. The results obtained show that the temperature, and hence, the resistance of the individual suspended microbridge sensor device, depends on the surrounding gas and its thermal conductivity, while the sensitivity and power consumption depend on the properties of the constitutive metal. Moreover, the electrode resistance is proven to be linearly dependent on the applied voltage.

## 1. Introduction

Air pollution is a major health hazard, being the reason for serious health problems in the long term, such as chronic and acute respiratory diseases including asthma, cardiovascular problems, stroke, lung cancer, and lower life expectancy [1,2]. In 2019, outdoor air pollution was reported to be the reason for 4.2 million premature deaths globally. This figure increases to an annual 6.7 million when combining the effects of household air pollution [2].

Gases are one type of contaminant that is present in the air. Examples include carbon dioxide (CO_2_) and ammonia (NH_3_). CO_2_ is a colourless, odourless gas [3]. Although normally produced by cellular respiration and naturally present in the atmosphere, its concentration has increased by 45 percent since the start of the Industrial Age to a current value of 412 parts per million [4], being a product of fossil fuel combustion. It leads to respiratory disorders and contributes to global warming [5]. On the other hand, NH_3_ is a colourless, highly irritating gas, with a suffocating odour [6]; it is widely emitted by agricultural activities, contributes to the formation of particulate matter through its reaction with acid gases (e.g., sulphuric and nitric acids) [7], and absorbs visible radiation, contributing to impaired atmospheric visibility and climate change [8].

As people are becoming more aware of their personal exposure to pollutants, the development of compact personal exposure monitoring devices plays a critical role in providing insights on air quality to guide their lifestyles, including mobility and heating habits. The first step in developing such devices consists of designing the sensor unit technologies required for data acquisition.

Different techniques have been explored for gas sensing over the years:

- Catalytic pellistors: Conventionally used for gas detection, these devices consist of an active bead, encasing a platinum coil and treated with a catalyst, to reduce the temperature at which the surrounding gas ignites. When the target gas burns, the active bead heats up and a resistance change proportional to the gas concentration is achieved. There have been microfabrication processing efforts to reduce their typically large power consumption [9,10,11].
- Acoustic techniques: These devices detect the change in the oscillation frequency of an oscillating (e.g., micromachined cantilevers) or piezoelectric material in the presence of a target gas. For instance, a sensitive and selective material can be used as a coating; the adsorption of the target gas on this coating results in a change in mass, and hence, the oscillating frequency [12]. Miniaturisation-compatible, low-power technologies include quartz crystal microbalance (QCM), surface acoustic resonators (SAR), and film bulk acoustic resonators (FBAR).
- Chemiresistive sensors: These devices detect gases through a chemical reaction between the sensing layer and the gas. Metal oxides have traditionally been used for the sensing layer, and their main advantages are their selectivity to some oxidising or reducing gas species, small size, high sensitivity, and fast response [13]; however, these sensors usually require heating [14], and research is still being conducted to reduce their power consumption [11]. Recently, 2D materials, mainly graphene and graphene oxide, have been explored as sensing layers, and their effective gas detection and discrimination have been proven at room temperature [15].
- Field-effect transistors (FETs): These devices have been proven to detect hydrogen, which, when adsorbed on the metallic gate, produces a change in the transistor threshold voltage. Other implementations using FETs or MOSFETs (Metal Oxide Semiconductor FETs) are also possible [12,16].
- Electrochemical sensors: These devices exist in two types: potentiometric and amperometric. Potentiometric sensors detect the change in the differential voltage between a working electrode and a reference electrode in the presence of a target gas. Amperometric sensors, on the other hand, are based on an enzyme-catalysed ion exchange between a working electrode and a counter-electrode, which results in a current change in the function of the target gas concentration [12,17].
- Optical sensors: These devices operate based on the principle that gases are able to absorb light in the infrared region, with the absorption profile being specific to each gas [12].

However, as low power, low cost, and a fast response time are required, there has been an increased focus on thermal conductivity detectors (TCDs) [18], made of electrically conductive microbridges in a thin SiN membrane suspended over a cavity, whereby a change in electrical resistance is observed by stimulating the device with a short electrical current pulse producing rapid Joule heating of the device, which is dependent on the thermal conductivity of the gas in question. Ultra-low-power TCDs have been demonstrated whose fabrication is compatible with the industrial manufacturing capabilities available for MEMS devices [11].

This paper explores the operation principle behind TCD sensors and uses results from finite element modelling to guide the design and to reveal insights into the impacts of different microbridge materials and their applicability to gas sensing. A metallic electrode is separately exposed to air, CO_2_, and NH_3_, and its behaviour, in terms of the required input voltage, temperature, and current/resistance is observed and analysed, both in steady-state and transient modes.

## 2. Materials and Methods

### 2.1. Functional Mechanisms of TCD Gas Sensors

Electrical resistance linearly depends on temperature, according to the following equation:

(1) $$R=R_{0}\left(1+\alpha \cdot ∆T\right)$$

where R is the resistance value at temperature T, $R_{0}$ is the resistance at the initial temperature $T_{0}$, ∆T is the temperature change, and α is the temperature coefficient of resistivity (TCR) at temperature $T_{0}$, which is a temperature-dependent characteristic property of the material.

The sensor, which is a metallic wire or conductive track on a thin membrane suspended over a cavity, is activated by flowing a current through it and heating it up to a certain temperature, characterised by a specific resistance value: this is the Joule heating effect. However, due to the thermal conductivity of gases, heat dissipates from the sensor into the surroundings, which changes its temperature, and therefore, resistance. The temperature can be kept constant by keeping a constant current. The presence of a different gas around the sensor results in a different heat dissipation rate, and hence, different temperature and resistance values. So, depending on the surrounding gas, the sensing element will have a different temperature, and therefore, resistance, which is, in this case, the output signal [11].

It is worth noting that Equation (1) is only valid when Joule heating is negligible, i.e., when the temperature is constant along the resistor length. In cases like ours, where the Joule effect causes the temperature to be distributed in a non-uniform fashion along the electrode, with significant thermal gradients at the end points or bonding pads [19], Equation (1) cannot be directly applied. Therefore, finite element modelling (FEM) was used to simulate the heating effect and extract the corresponding resistance values. A simulation was developed and will be discussed in the following sections.

### 2.2. FEM Workflow

A finite element simulation model of the microbridge device in a volume of air in the presence of gases with different thermal conductivity and its influence on electrical resistance when subject to an electrical current was studied. COMSOL Multiphysics® (v. 6.0. COMSOL AB, Stockholm, Sweden) was the simulation tool of choice for FEM in this work.

The modelling workflow in COMSOL [20] is composed of the following steps:

- Building the geometry.
- Assigning a material to each part of the geometry.
- Choosing suitable physics and boundary conditions—COMSOL also has Multiphysics nodes, which allows us to combine two physics and observe their combined effects.
- Meshing the geometry.
- Choosing a study and simulating the model.
- Postprocessing the results.

### 2.3. Geometry and Materials

**Figure 1:**
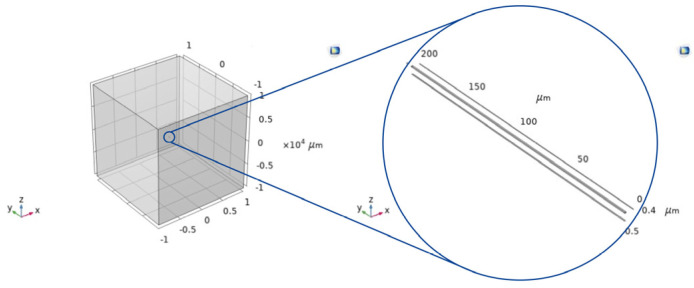
Full geometry of the electrode placed at the centre of the gas block (**left**)—the electrode is very small compared to the gas volume. Electrode-only zoom-in (**right**).

A metallic electrode of dimensions 1 × 200 × 0.68 μm, in a relatively big gas volume, was simulated. The geometry is shown in Figure 1. For the material selection, Material Switch nodes were adopted to simulate different material combination possibilities. Aluminium (Al), gold (Au), and tungsten (W), which are commonly used in MEMS sensors and therefore frequently used for heating elements [18], were used for the electrode. Air, CO_2_, and NH_3_ were used for the gas. Table 1 shows details of the properties of the metals.

**Figure 2:**
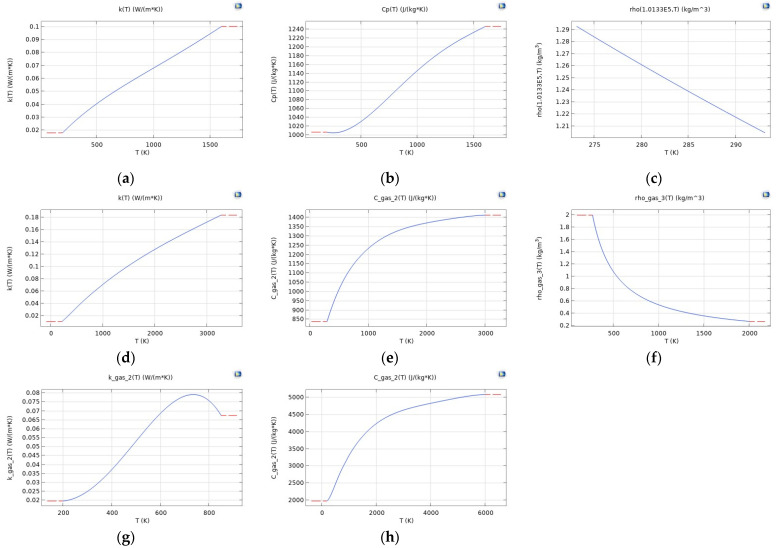
Thermal conductivity (**a**,**d**,**g**), heat capacity at constant pressure (**b**,**e**,**h**), and density (**c**,**f**) of air (**a**–**c**), CO_2_ (**d**–**f**), and NH_3_ (**g**,**h**). NH_3_ has a constant density of 0.73 kg/m^3^ (not plotted).

The properties of the gases, however, are defined as a function of temperature. Figure 2 plots the thermal conductivity, heat capacity at constant pressure, and density for all 3 gases.

### 2.4. Physics

In order to study the Joule effect, two physics nodes were needed: Electric Currents and Heat Transfer in Solids and Fluids. These two physics were then combined into a Multiphysics node to study the Electromagnetic Heating.

The Electric Currents physics [21] was applied to the electrode only. Linearized resistivity was selected for the Conduction model to simulate the linear resistance change as a function of temperature. The simulation uses the following equation, which is practically the same as (1) presented earlier, knowing that conductivity is the inverse of resistance:

(2) $$\sigma =\frac{1}{\rho_{0}\;\left(1+\alpha \;\left(T-T_{ref}\right)\right)}$$

where

$T_{ref}$: reference temperature, considered to be 20 °C (293.15 K);
$\rho_{0}$: reference resistivity at $T_{ref}$;
α: resistivity temperature coefficient (TCR).

These values were taken from [22,23] and are stated in Table 1.

Two boundary conditions were added: electric potential and ground, applied to opposite ends of the electrode. A Parametric Sweep was used to vary the electric potential in the range 0–0.4 V, with steps of 0.01 V.

The heat transfer study was conducted using the Heat Transfer in Solids (electrode) and Fluids (gas domain) [24] interface. A fixed-temperature boundary condition set to 20 °C was applied on the outside walls of the gas volume to model the cooling effect.

### 2.5. Meshing

Particular care was taken in defining the size and types of mesh elements in line with the device geometry with a small-sized electrode compared to the gas volume, needing computationally effective models that avoid the risk of misrepresenting the electrode. For this, the electrode and the gas volume were meshed separately. The gas was meshed using normal-sized free tetrahedral elements, while a Swept mesh was used for the electrode. A Mapped node was used to specify the mesh element to be one of the small electrode faces—a quadrilateral element—which was then Swept and converted into 3D hexahedral elements in the electrode.

### 2.6. Study

First, a time-dependent study was conducted over a time period of 10 s with 1 s intervals. As a steady state was almost reached by the first second, shorter simulations and granular time steps were adopted in the second instance, and the results are discussed in Section 3.4.

## 3. Results and Discussion

### 3.1. Centre Temperature

A 3D cut point of coordinates (0.5, 100, 0.68 μm), located at the centre of the upper face of the electrode, was selected. The highest temperature was reached at the centre of the electrode, and temperature was plotted at this cut point.

**Figure 3:**
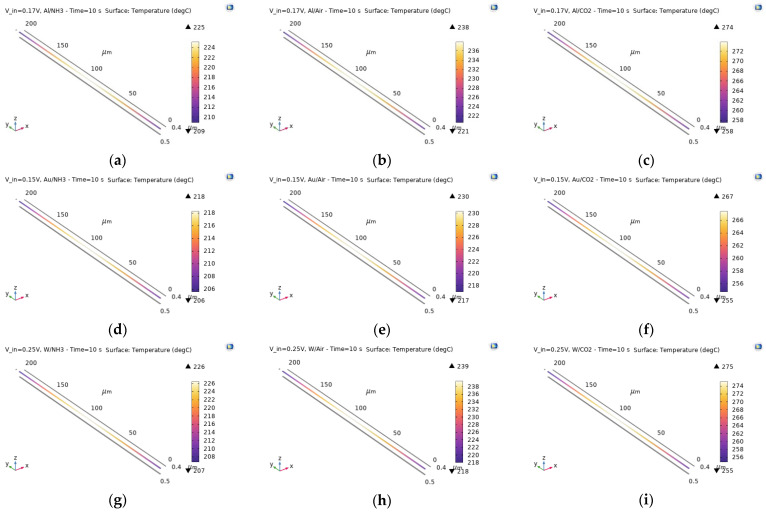
Temperature distribution in the electrode for the different metal–gas combinations at the last simulation time of 10 s. The metals used are Al (**a**–**c**), Au (**d**–**f**), and W (**g**–**i**) with respective input voltages of 0.17 V, 0.15 V, and 0.25 V, while the gases are NH_3_ (**a**,**d**,**g**), air (**b**,**e**,**h**), and CO_2_ (**c**,**f**,**i**).

**Figure 4:**
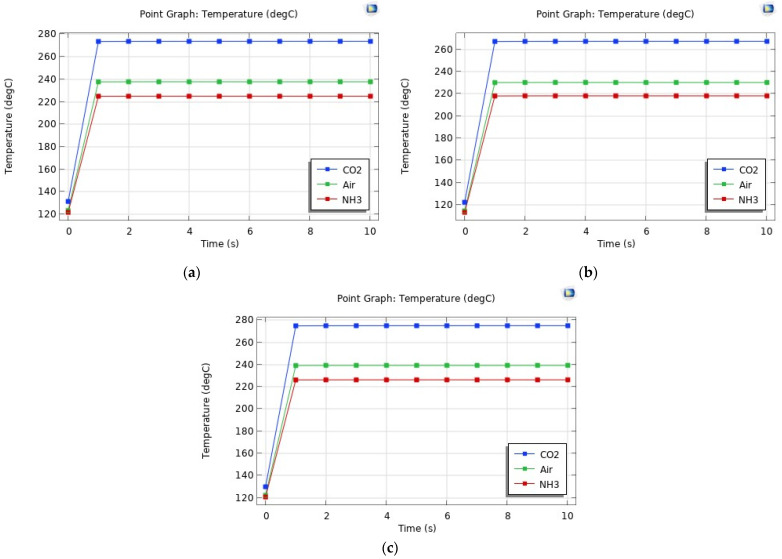
Graphs of the temperature at the centre of the electrode as a function of time with all 3 gases for Al (**a**), Au (**b**), and W (**c**).

Looking for temperatures over 200 °C for all metal–gas combinations, the required input voltages were found to be 0.17 V, 0.15 V, and 0.25 V for Al, Au, and W, respectively. Figure 3 shows the temperature distribution plots in the electrode for the different material combinations at the final time (10 s), while Figure 4 presents the graphs of the temperature as a function of time.

Two important observations can be made based on these results:

- Au, Al, and W, respectively, require increasing input voltages for the electrode to reach the same temperature range.
- NH_3_ results in a lower electrode temperature, while CO_2_ results in a higher one, compared to air, regardless of the electrode material.

The first observation is due to the different resistivities associated with the different materials. The cuboid-like shape of the electrode allows us to easily calculate its resistance using the following formula:

(3) $$R=\rho \;\frac{L}{A}=\rho \;\frac{L}{Wt}$$

where R is the resistance; ρ is the resistivity of the electrode material; L, W, and t are, respectively, the length, width, and thickness of the electrode; and A is the cross-sectional area.

In the current situation, L = 200 μm, W = 1 μm, and t = 0.68 μm. The resistivities of the metals are mentioned in Table 1. Therefore, the cold resistance of the electrode at 20 °C is 7.059 Ω for Au, 7.794 Ω for Al, and 16.471 Ω for W.

The heating power associated with the Joule effect depends on the square of the voltage and the inverse of the resistance. Therefore, in order to obtain the same heating level (i.e., temperature), higher voltages are required for increasing resistances, which corroborates the first observation.

**Figure 5:**
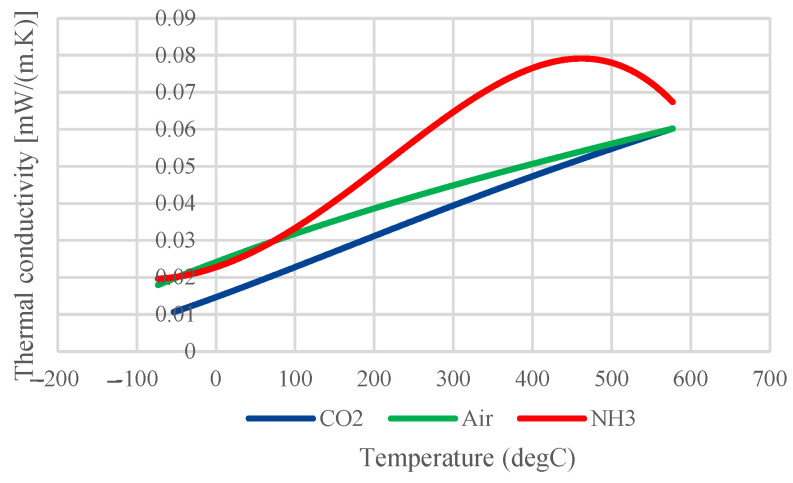
Thermal conductivity of air, NH_3_, and CO_2_ as a function of temperature (data from COMSOL).

Looking into the second observation, the different response is directly related to the thermal conductivity of the gases in question, which is plotted as a function of temperature in Figure 5. In the considered temperature range, NH_3_ has the highest thermal conductivity, while CO_2_ has the lowest. In fact, the higher the thermal conductivity, the more heat dissipation out of the electrode will occur, which results in a lower electrode temperature [25].

From the second observation, the different electrode temperatures in the presence of different gases implies a change in resistance. This will be further discussed in Section 3.2 below, based on the measured values of electrical current as a function of the applied voltage values.

### 3.2. Current and Resistance

The finite element model developed in COMSOL allows us to derive the current density J (A/m^2^) as the simulation result under different boundary conditions and input voltages. In order to find the current I (A), the surface (double) integration of the normal current density is computed on the electrode face where the potential is applied (of area A).

At the same applied voltages mentioned in A, the current is observed as a function of time. In a steady state, different current levels are obtained depending on the type of surrounding gas. Knowing that the input voltage is constant, a difference in resistance is therefore detected.

From the steady-state currents obtained, resistance can be calculated using Ohm’s Law. The resistance values obtained for the different metal–gas combinations are reported in Table 2, along with the percentage difference in resistance in the presence of a gas, relative to air, %Δgas, which can be seen as a measure of sensitivity to a specific gas, and is given by:

(4) $$\%∆gas=\left|1-\frac{R_{gas}}{R_{air}}\right|\times 100$$

**Figure 6:**
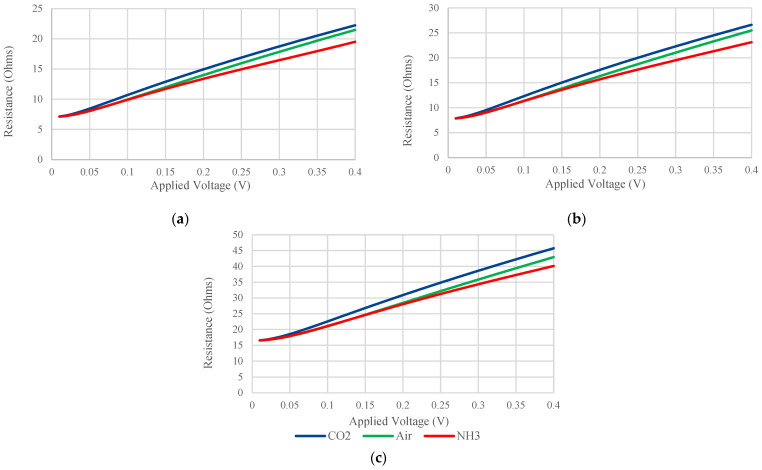
Electrode resistance as a function of the input voltage in the presence of each of 3 gases (CO_2_, air, and NH_3_) for all 3 electrode materials (Al (**a**), Au (**b**), and W (**c**)).

Three observations can be made on these simulation results:

- The resistance values in Table 2 are higher than the cold resistances mentioned in Section 3.1 due to the heating effect.
- The presence of NH_3_ results in a lower resistance than air, while CO_2_ results in a higher resistance. This is related to the second observation made in Section 3.1 and the linear relationship between temperature and resistance.
- The percentage resistance difference %Δgas is highest for W, followed by Al and Au, respectively, regardless of the surrounding gas. This is due to TCR of the metal (Table 1): a metal with a higher TCR shows higher sensitivity, as explained in [18].
- The current no longer increases linearly with the input voltage because the resistance is changing. Plotting the resistance as a function of the input voltage, the graphs in Figure 6 are obtained.

Some observations can be made from Figure 6:

- Once again, it is confirmed that CO_2_ results in higher resistance than air, while NH_3_ produces lower resistance.
- There is a linear relationship between resistance and the applied voltage.
- At low voltages, the difference in resistance between the 3 gases is minimal. Therefore, in order to obtain better sensor performance, it is recommended to use input voltages over 0.15 V for Al and Au, and 0.2 V for W. The higher the input voltage, the more obvious the difference in the resistance values; but in actual devices, it is important to consider the fuse effect so as not to blow the electrodes.

### 3.3. Voltage and Temperature

**Figure 7:**
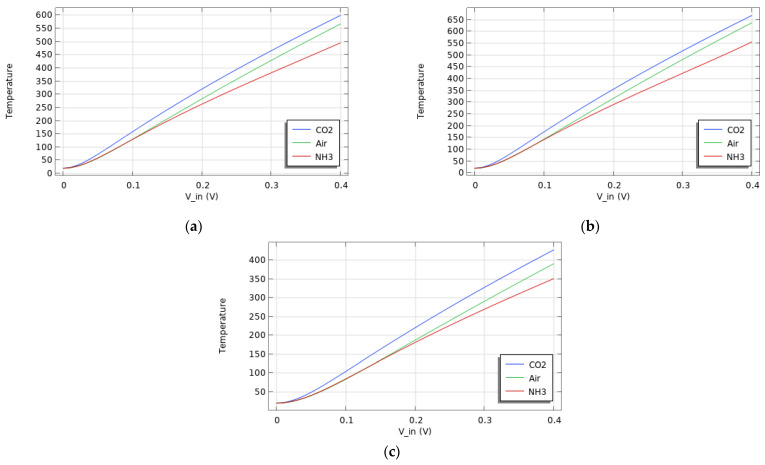
Electrode centre temperature as a function of the input potential for all 3 gases (CO_2_, air, and NH_3_) and electrode materials (Al (**a**), Au (**b**), and W (**c**)).

In Section 3.2, a linear relationship is observed between resistance and applied voltage. There is a linear relationship between resistance and temperature as well. In fact, temperature also depends linearly on the input voltage, as shown in Figure 7, where the temperature is plotted as a function of the applied potential.

It can be noticed that at a set input voltage, CO_2_ results in a higher electrode temperature than air, while NH_3_ results in a lower temperature, which correlates with observations made in the previous section, where CO_2_ and NH_3_ were associated with higher and lower resistances, respectively.

### 3.4. Transient Behavior

Using more granular time steps for the simulation, the centre temperature is found to reach around 90.41% of its steady-state value (at 10 s) after 0.2 ms, and 98.71% after 0.01 s.

**Figure 8:**
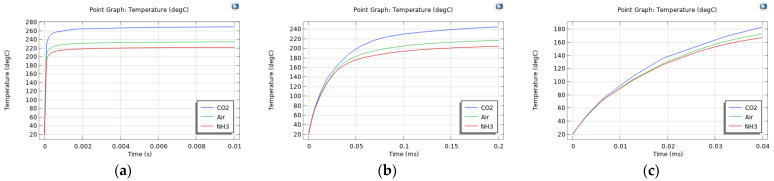

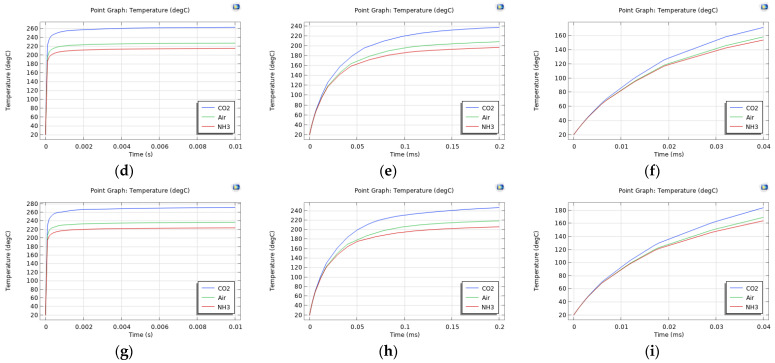
Electrode centre temperature for Al (**a**–**c**), Au (**d**–**f**), and W (**g**–**i**) over 0.01 s (**a**,**d**,**g**), 0.2 ms (**b**,**e**,**h**), 0.04 ms (**c**,**f**,**i**) timeframes.

Temperature differences in the presence of different gases are visible as early as 0.04 ms, when the temperature has reached 69.83% of its steady-state value; the associated resistance values are listed in Table 3 and can be compared with the steady-state resistances in Table 2. Though the temperature does not increase linearly over time (0.04 ms plot in Figure 8 and different ratios for T and R in Table 3), the hypothesis suggesting that different slopes can be observed for different gases is rejected.

## 4. Conclusions

In this work, an FEM simulation was used to investigate the sensing mechanisms associated with TCD gas detectors, based on the fundamental principles of thermal conductivity characteristics of different gas molecules, which shows potential for application in the continuous monitoring of toxic and polluting gas species in air. Some relevant conclusions can be drawn:

- The material resistivity, which determines the electrode resistance, influences the input voltage level required for the electrode to reach a specific temperature: higher voltages are required for higher resistances/resistivities, and vice versa.
- The gas thermal conductivity, which determines the amount of heat dissipation, influences the electrode temperature. At a set input voltage, and regardless of the electrode material, a gas with higher thermal conductivity results in a lower electrode temperature, and vice versa.
- Building upon the previous point, the current flow in the electrode, and hence, its resistance, depend on its temperature. A gas with higher thermal conductivity results in lower electrode resistance and a higher current.
- The higher the TCR of the electrode material, the higher the sensitivity.
- There is a linear relationship not only between resistance and temperature, but also between resistance and voltage, and temperature and voltage.
- Based on the above, W is the best electrode material in terms of sensitivity, but it also induces higher power consumption (higher resistance and required input voltage). Au, on the other hand, has the lowest sensitivity, but draws the least power.

Given the foundational aspects of the problem presented, the intrinsic limitations of the finite element analytical model, and the findings reported as a result of the numerical simulations, further experimental work is required to assess the behaviour of TCD sensors in real-world working conditions and confirm the accuracy of the simulation results reported.

## Figures and Tables

**Table 1:** Material properties of selected metals for microbridges in TCD fabrication.

Material Property	Al	Au	W
Heat capacity at constant pressure [J/(kg·K)]	904	129	132
Density (kg/m^3^)	2700	19300	19350
Thermal conductivity [W/(m·K)]	237	317	174
Relative permittivity	1.55	6.9	1
Reference resistivity (Ω·m)	2.65 × 10^−8^	2.40 × 10^−8^	5.60 × 10^−8^
Resistivity temperature coefficient TCR (1/K)	0.00429	0.0034	0.0045
Reference temperature (K)	293.15	293.15	293.15
Electrical conductivity (S/m)	3.55 × 10^7^	4.56 × 10^7^	2.00 × 10^7^
Coefficient of thermal expansion (1/K)	2.31 × 10^−5^	1.42 × 10^−5^	4.50 × 10^−6^
Young’s modulus (Pa)	7.00 × 10^10^	7.00 × 10^10^	4.11 × 10^11^
Poisson’s ratio	0.35	0.44	0.28

**Table 2:** Resistance values (in Ohms) for the different metal–gas combinations.

	CO_2_	Air	NH_3_	%ΔCO_2_	%ΔNH_3_
Al	16.088	14.872	14.461	8.176	2.765
Au	12.887	11.991	11.709	7.466	2.357
W	34.845	32.170	31.261	8.314	2.827

**Table 3:** Electrode centre temperature and resistance after 0.04 ms, and ratios to their respective values at 10 s, for the different metal–gas combinations.

Material	Gas	T_0.04ms_ (°C)	T_0.04ms_/T_10s_ (%)	R_0.04ms_ (Ω)	R_0.04ms_/R_10s_ (%)
Al	Air	173.34	72.89	12.81	86.14
	CO_2_	183.83	67.14	13.19	82.00
	NH_3_	167.59	74.47	12.63	87.35
Au	Air	158.51	68.84	10.33	86.12
	CO_2_	171.86	64.29	10.66	82.71
	NH_3_	154.04	70.59	10.22	87.33
W	Air	169.36	70.76	27.28	84.79
	CO_2_	184.17	66.96	28.44	81.61
	NH_3_	164.18	72.53	26.93	86.13

## Data Availability

The data presented in this study are available on request from the corresponding author.
